# Adolescent and Young Adult Initiated Discussions of Advance Care Planning: Family Member, Friend and Health Care Provider Perspectives

**DOI:** 10.3389/fpsyg.2022.871042

**Published:** 2022-06-08

**Authors:** Sima Z. Bedoya, Abigail Fry, Mallorie L. Gordon, Maureen E. Lyon, Jessica Thompkins, Karen Fasciano, Paige Malinowski, Corey Heath, Leonard Sender, Keri Zabokrtsky, Maryland Pao, Lori Wiener

**Affiliations:** ^1^Center for Cancer Research, National Cancer Institute, Bethesda, MD, United States; ^2^Children’s National Hospital, Washington, DC, United States; ^3^Dana-Farber Cancer Institute, Boston, MA, United States; ^4^Cook Children’s Medical Center, Fort Worth, TX, United States; ^5^Children’s Hospital Orange County, Orange, CA, United States; ^6^National Institute of Mental Health, Bethesda, MD, United States

**Keywords:** AYA family, friends, HCP, adolescent and young adult, advance care planning, EoL discussions, Voicing My CHOiCES, communication

## Abstract

**Background and Aims:**

End-of-life (EoL) discussions can be difficult for seriously ill adolescents and young adults (AYAs). Researchers aimed to determine whether completing *Voicing My CHOiCES* (VMC)—a research-informed advance care planning (ACP) guide—increased communication with family, friends, or health care providers (HCPs), and to evaluate the experience of those with whom VMC was shared.

**Methods:**

Family, friends, or HCPs who the AYAs had shared their completed VMC with were administered structured interviews to assess their perception of the ACP discussion, changes in their relationship, conversation quality, and whether the discussion prompted changes in care. Open-ended responses underwent thematic analysis.

**Results:**

One-month post-completion, 65.1% of AYA had shared VMC completion with a family member, 22.6% with a friend, and 8.9% with an HCP. Among a sample of respondents, family (47%) and friends (33%) reported a positive change in their relationship with the AYA. Participant descriptions of the experience fell into five themes: positive experience (47%), difficult experience (44%), appreciated a guide to facilitate discussion (35%), provided relief (21%), and created worry/anxiety (9%). Only 1 HCP noted a treatment change. Family (76%), friends (67%), and HCP (50%) did not think the AYA would have discussed EoL preferences without completing VMC.

**Conclusions:**

VMC has potential to enhance communication about ACP between AYA and their family and friends, though less frequently with HCPs. Participants reported a positive change in their relationship with the AYA after discussing VMC, and described experiencing the conversation as favorable, even when also emotionally difficult.

## Introduction

The number of AYAs living with serious illnesses such as cancer is growing globally (Viner et al., 2011; Cohen and Patel, 2014; GBD 2019 Adolescent Young Adult Cancer Collaborators, 2022). In 2020, an estimated 90,000 adolescents and young adults (AYA) were diagnosed with cancer in the United States (Haines et al., 2021). For many, death is an inevitable outcome of their disease, making advance care planning (ACP) a critical component of care (Brown and Sourkes, 2006; DeCourcey et al., 2021). It is difficult for AYAs, their families, and providers to think about or talk about death and dying. The presence of a life-threatening illness adds a multitude of challenges to what is already a difficult period of life, when emerging adults strive to define themself outside of the context of their family and envision their own future (Brown and Sourkes, 2006). Further, disease burden at this age can negatively impact financial security, body image, educational and work trajectories, relationships with spouse/significant other, and plans for having children (Maslow et al., 2011; Warner et al., 2016; Jin et al., 2017). In addition, young adults with advanced cancer have reported significant psychological distress in the form of grief (Jacobsen et al., 2010) and suicidal ideation (Walker et al., 2008).

Effective end-of-life (EoL) discussions are critical for AYAs, especially in the event of disease progression or a poor prognosis, given both the medical challenges and psychosocial risk factors involved (Sansom-Daly et al., 2020). ACP documents and advance directives provide patients with the opportunity to express their preferences for care. These directives can help families and health care agents make informed decisions, alleviate distress (Mack et al., 2005), avoid decisional regret (DeCourcey et al., 2019; Lichtenthal et al., 2020), and potentially improve the patient’s quality of life by respecting their religious, cultural, and familial values and beliefs (Jankovic et al., 2008; Barfield et al., 2010; Kane et al., 2011; Wiener et al., 2012). Families have expressed significant interest in ACP, with parents indicating that the opportunity for these discussions has been a poorly met need (Durall et al., 2012; Lotz et al., 2013; DeCourcey et al., 2019; Hein et al., 2020; Orkin et al., 2020). Research has shown that parents of seriously ill children desire earlier, ongoing opportunities to address ACP with their child’s providers (DeCourcey et al., 2019; Orkin et al., 2020). However, many pediatric providers report a lack of ACP communication training (Dellon et al., 2010; Durall et al., 2012; Lotz et al., 2013; Heckford and Beringer, 2014). Unfortunately, when ACP discussions do take place, they often occur too late and typically during an acute clinical crisis, when there is insufficient time to consider individual goals and values (Davidson et al., 2007; Brudney, 2009; Durall et al., 2012; Snaman et al., 2020; Pennarola et al., 2021).

The literature has clearly demonstrated the need and desire for ACP intervention in this population (Weaver et al., 2015; Kirch et al., 2016), as well as the barriers to initiating ACP (Wolfe et al., 2008; Smith et al., 2012; Kassam et al., 2013; Pinkerton et al., 2018). Studies have shown that less than 3% of AYAs participate in EoL planning conversations without clinician prompting (Lyon et al., 2004, 2014; Liberman et al., 2014; Carr et al., 2021). Data exploring the experience of the family members, friends, and health care providers who are involved in ACP conversations with AYA are limited. Adolescents and families who participated in family-centered ACP found the conversations to be worthwhile (Dallas et al., 2016), notably with a greater understanding of EoL wishes (Madrigal et al., 2017). In a robust multi-site, assessor-blinded, parallel-group, randomized control trial (*FACE pACP*), ACP surrogates were eight times more likely than controls to have an excellent understanding of adolescent patients’ treatment preferences (Lyon et al., 2018). In another ACP trial, families had more positive appraisals of their caregiving, than families who did not have these conversations (Baker et al., 2020).

Novel tools and interventions are needed to facilitate ACP discussions between AYA and their family members, friends and HCPs (Snaman et al., 2020). *Voicing My CHOiCES* (VMC), a research informed ACP guide (Wiener et al., 2012; Zadeh et al., 2015), has been shown to both decrease anxiety around EoL planning and enhance communication with both family members and friends (Wiener et al., 2021). This study adds to the literature by providing the perspectives or outcomes on behalf of the family member, friend, or HCP post completion of an ACP document by an AYA. In this study we aimed to gain understanding of the experience of the family member, friend, or HCP pertaining to the ACP discussion, changes in their relationship, conversation quality, and whether the discussion prompted changes in care.

## Materials and Methods

### Study Recruitment and Enrollment

As part of a larger study, AYAs aged 18–39 years receiving cancer-directed therapy or treatment for another chronic medical illness at one of seven study sites were enrolled on a larger study examining psychosocial outcomes after completing VMC. For this sub-study, participants included the family members, friends, or HCPs with whom the AYAs initiated a conversation with about their ACP preferences following completion of the VMC guide. Sub-study participants were contacted by phone, with permission from the AYA by whom they had been nominated. The NIH Institutional Review Board approved this protocol, and the study was then approved by the IRB at each of the participating sites. Data was collected between 2015–2019.

### Study Procedures

AYAs were contacted one-month post-VMC completion to seek permission to contact any family member, friend, or HCP the AYA had shared preferences with. Informed consent was subsequently obtained and each participant was then administered a one-time structured interview, including both quantitative and open-ended questions. Development of the interview was based on shared clinical expertise of the primary study team, familiarity with the VMC guide, and knowledge of the relevant literature and gaps therein (Wiener et al., 2012, 2021; Dallas et al., 2016; Sansom-Daly et al., 2020). Specifically, the interview assessed the communication they had about ACP with the AYA, as well as perceived changes in their relationship, the quality of the conversation, and whether changes in care were made following the discussion. Interviews were conducted either in person or by phone and responses were written verbatim by the interviewer. No audio or video recordings were collected. Each interview took approximately 15 min to complete. See Supplemental Data Sheet 1 for the interview guide.

Interviews were conducted by a trained study team member, including psychologists, social workers, nursing study coordinators, or graduate students. Procedure training consisted of an in-person, virtual or phone session with the sponsor site (SZB or LW) where a training manual was reviewed in detail and sample case scenarios were discussed.

### Analysis

Responses to open-ended questions were analyzed using a realist approach to inductive thematic analysis (Braun and Clarke, 2006; Maxwell, 2012). Coders (SZB, AF, LW) independently read and re-read the data, identifying initial codes, capturing novel content, and searching for potential themes (Braun and Clarke, 2006; Miles et al., 2014). The coders then met as a group to review codes, and to examine, refine and define themes (Macqueen et al., 1998). Discrepancies were resolved through consensus discussion. Free-text responses were then coded in parallel (Malterud, 2001). The authors reviewed and discussed the findings and summarized the data.

## Results

One month after completing the baseline measure and reviewing the VMC tool, 129 participants answered the follow up questions about talking with family members, 124 about talking with friends, and 124 answered the question about talking with HCP. Overall, 84 (65.1%) of participants had shared what they wrote in VMC with a family member and 11 (8.9%) shared with an HCP (Wiener et al., 2021). Twenty-eight participants (22.6%) shared what they wrote in VMC with a friend. Of those with whom document completion was shared, we interviewed 40 (47.6%) family members, 6 (21.4%) friends, and 5 (45.5%) providers about their experience with this conversation. Of note, three interviews (two family members and one provider) were discontinued when the participant indicated the AYA had not shared what they had written in VMC. The remaining analyses are based on interviews with the 48 participants who engaged in such a conversation, according to both the AYA and the study participant.

### ACP Discussions Had Pre- and Post-VMC

For 17 (42.5%) of the 40 family members interviewed, their first ACP conversation was held post-AYA VMC completion. Of the friends interviewed, 4 of the 6 friends (66.7%) were from AYAs who first spoke to their friend after VMC. Of the five providers interviewed, 3 (60%) were from AYAs who only spoke to their HCP about ACP post-VMC. Twenty-nine (76.3%) family members, 4 friends (67%) and 2 HCP (40%) did not think the AYA would have talked to them about their EoL preferences without the study. When participants were asked *“Can you tell me what part of the advance care planning process [the patient] shared with you,”* four themes were revealed: preferences on comfort/support, care preferences when critically ill, planning for remembrance, and care/concern for others after death. Sample responses are provided in Table 1. One HCP (20%) noted a treatment change following the discussion (e.g., medication changes for symptom management).

**TABLE 1:** Part of ACP shared with participant post-VMC completion.

Shared component of ACP	Coding definition	Participant responses
Preferences on Comfort/Support	Discussed ways to provide comfort and support, including how to manage visitation	*“Doesn’t want a lot of people visiting if very sick.”* *“He shared how he wants to be treated when not feeling well and what’s comforting to him, even in his room.”* *“A list of who could visit, and “no one can cry.” She wants mental health professionals on call for visitors.”* *“Emotional support if things don’t go well. Someone to call or be there for him if he gets really sick”*

Care Preferences When Critically Ill	Discussed who will make care decisions if they are unable to and what kind of care preferences they have, including life support options and where they want to be at the end of life	*“If he would get worse, he would want his older brother to make life support decisions for him. And me too*…*He shared that he did not want his dad to make decisions because his dad would never say no to life support, and he does not want to live on life support unless it is reversible.”* *“The chain of command if something happened to him.”* *“We went over worst-case scenarios. She read through it with me. It brought up issues we hadn’t talked about before. Now, I know what she wants.”* *“She said if she was connected to a machine for surviving, she would accept that for a short time but if they found that there was no improvement, and she would not get better she would not be connected to these machines.”* *“That he would want to be home at the end.”*

Planning for Remembrance	Communicated thoughts on after death and funeral planning, how to distribute/donate belongings	*“What she’d like at a funeral, music, and how she would like to be remembered.”* *“Would not want an open casket. Wants a celebration of life.”* *“The section “How I wish to be Remembered.” The main part that was shared was regarding what he would be leaving behind and to whom he would leave certain things* (*i.e., his personal belongings).”* *“Details about things she had thought about but hadn’t spoken to us about- especially after death* (*Belongings, celebration, cremation, donate her body, where her ashes should go)”* *“What she wants after she dies. No open casket which surprised me. “If I can’t see then, they shouldn’t be able to see me” Hospice at home.* *“That she would want her ashes going out to sea”*

Care/Concern for Others After Death	Expressed concern for the care of others left behind after their death	*“The will for what happens after she dies. We talked about how the stuff with the kids was missing. We wanted to be able to say where they go, who takes care of them, how finances get to them.”* *“His concern about his little sister.”* *“It was very focused on her daughter understandably. I wish she had written more specific details of what to do* (*with her daughter, what traditions to continue etc.).”*

### Changes in Relationship

Forty-seven percent of family members (*n* = 19) and 33% of friends (*n* = 2) reported a change in their relationship with the AYA following the discussion. If a change in their relationship was reported, participants were then asked to describe the change. Four themes were found: the conversation opened lines of communication, increased feelings of closeness, learned something that was important, and changed view of character (i.e., how the AYA thinks, feels or copes). Sample responses are provided in Table 2.

**TABLE 2:** Perceived changes in relationship post-VMC discussion.

Theme	Coding definition	Sample response
Opened lines of communication	Created an opportunity for discussion, broke down barriers	*“[study participation] cracked open that door and allowed him to express things he was probably thinking about since diagnosis and questions about whether he will survive. That barrier has now been broken down and we feel we can probably talk about anything.”* (*family member)* *“We were avoiding the questions before- broke down barriers.”* (*family member)*

Increased feeling of closeness	Enhanced sense of connection between individuals	*“I feel closer to him.”* (*friend)* *“These conversations do bring you closer.”* (*family member)*

Learned something that was important	Gained knowledge about AYAs preferences and/or values	*“Few things I didn’t know* (*his cross/chain) and how important those things are to him.”* (*family member)* *“I realize more how much she needs me and relies on me.”* (*family member)*

Changed view of character	Changed view of how AYA thinks, feels, copes, behaves	*“I look at her as fearless and with more respect”* (*family member)* *“She grew so much from completing this”* (*family member)*

### How the ACP Conversation Was Experienced

Family members, friends and HCPs were also asked, “*Can you tell us what this experience was like for you?”* What emerged illustrated both benefit and burden. Themes that represented benefit included the conversation being a positive experience, appreciating a guide to facilitate a deeply honest conversation and the conversation provided relief. Themes that represented a burden included experiencing the conversation as emotionally difficult and that it created worry/anxiety. While some participants described a sense of burden from the discussion (e.g., difficult experience or created worry/anxiety), the majority described benefit (e.g., positive experience, appreciated guide, provided relief). Of those participants who reported a burdensome experience most indicated finding benefit despite the burden (e.g., painful to have the discussion but grateful to know what their EoL preferences are). Select participant responses are provided in Table 3.

**TABLE 3:** Participant perceptions of the ACP discussion.

Theme	Coding definition	Sample quote
Positive experience	Experience was found to be helpful or beneficial	*“It is really good to know what he wants. allowed me to see where she was coming from, not just as a patient, but also as a person. Created open space for her to discuss difficult topics with me.”* (*provider)* *“This was a very intimate conversation, it opened doors for us. It brought trust between us that we can now talk more openly. First time we could do this. It was a true gift to me as his mom, to our relationship, and to our whole family.”* (*family member)*

Difficult experience	Experience was burdensome, emotionally, for the participant	*“Very hard. I choked up but had to remember this is not happening now, and we need to talk about all of it.”* (*family member)* *“Very intense. I didn’t expect to get so sad. It’s so much more thorough than previous conversations.”* (*Family member)*

Voicing My CHOiCES provided an opportunity for discussion	Benefit of the actual ACP guide was endorsed	*“It was nice for this to be available and not just all on me to remember everything she said. Makes it easier on me. And it provides us a vehicle to expand on her thoughts and preferences if she doesn’t respond to this next treatment.”* (*family member)* *“The document is great because it provides a medium for the conversation. It is also great that it allows conversations to happen when we aren’t in crisis, this makes it easier to talk about these things.”* (*family member)*

Provided relief	Participant noted feeling better having had the conversation	*“I was glad he had an opportunity to talk to someone other than me. Handling this better at his home center. Glad he’s not holding things in. I am more relaxed that he is less stressed. I never want to think about these issues, but I am so relieved since we are both thinking about “it” and neither knew how to broach the subject.”* (*family member)* *“Relieved because we were not talking about what was happening.”* (*family* *member)*

Created worry/anxiety	Experience contributed to psychosocial distress	*“There is a lot of pressure/burden on me to be her #1 and her parents aren’t even on the list* (*or aren’t prominent on the lists of people). It is kind of stressful to think she needs me that much.”* (*friend)* *“A lot on me with the family dynamics– I’ll be the one to do a lot. Sad/scary to think about going down that path.”* (*Family member)*

## Discussion

ACP discussions have been associated with a range of positive outcomes, including increased congruence between treatment preferences expressed by AYAs and their caregivers and increased likelihood that these preferences will be honored at the EoL. Yet, AYAs and their caregivers find it difficult to engage in these conversations (Jimenez et al., 2018). For the majority of study participants, completing an age-appropriate ACP guide prompted a first conversation regarding EoL preferences with a family member. To a lesser degree, it also prompted a first conversation with a friend. Notably, many of these conversations covered more than just EoL preferences. Participants described having deeply honest discussions about hopes, fears, and relationships. These findings support using VMC to enhance communication about EoL preferences, adding to the existing literature on the myriad benefits of such interventions (Feraco et al., 2016; Lin et al., 2020; Laronne et al., 2021).

In addition to families requiring assistance in navigating these discussions (Kenney et al., 2021), we found few AYAs shared what they wrote in VMC with their HCP. Challenges surrounding ACP conversations with HCPs include provider discomfort and a lack of training and resources (Dellon et al., 2010; Durall et al., 2012; Lotz et al., 2013; Heckford and Beringer, 2014). In fact, most family members and friends, as well as half of the HCP in our study, did not think the AYA would have talked to them about their EoL preferences without having completed VMC. This highlights the need for training on how to introduce ACP more comfortably with AYAs so that goals regarding current and future care can be addressed. While just one of five HCPs noted a treatment change following the discussion, these numbers may reflect the limited number of HCPs who the AYAs engaged in an ACP conversation. This is consistent with current literature suggesting patients and their family members will wait for the topic to be raised by their clinician (Clayton et al., 2005; Brighton and Bristowe, 2016). Research focused on training clinicians and preparing patients and families to engage in high-quality discussions using an age-appropriate ACP guide, like VMC, may help to achieve higher quality EoL care.

The specifics of what the AYA shared following completion of VMC was varied and included their preferences on mechanisms of comfort and support, how aggressively they would like to be treated if there was little to no chance of recovery, planning for how they would like to be remembered, and care/concern for others after death. These conversations were well received and described as being beneficial to both parties, despite being emotionally difficult to initiate. Similar to extant literature (Aldridge et al., 2017; Hein et al., 2018; Weaver et al., 2021), participants in the current study recognized that communicating important EoL care preferences can help prepare for future situations and create a pathway to goal-concordant EoL care. Critical longitudinal data is needed to assess whether communicated preferences were honored after an AYAs death.

Other benefits from these conversations were also reported. Half of family members and a third of their friends reported a change in their relationship with the AYA. Changes were all self-reported to be positive. Additionally, when describing the overall experience of talking about ACP with the AYA, participants again highlighted benefit despite also being seen as burdensome. Many participants spoke to the value of the ACP conversations and the relief in having the discussion despite the stress of thinking about worst-case scenarios. These findings can reassure family members and HCPs of advantages associated with these courageous conversations.

Some limitations are important to note. First, many family members (*n* = 44, 53.7%), friends (*n* = 6, 54.5%), and HCP (*n* = 5, 50%) who AYAs shared their VMC completion with were not interviewed. For some, the AYA wasn’t comfortable with having the researcher reach out to them, and for others the family member, friend, or HCP was unreachable or declined participation. Second, interviews were only conducted with English-speaking individuals. Therefore, we do not know if the themes that were identified would be different from the family members, friends and HCP who were not interviewed. Third, we contacted AYA participants 1 month after they completed VMC. We don’t know if a conversation occurred with a family member, friend, or HCP about what they wrote in VMC past this point. Fourth, demographic data was not collected on the contact participants, so it is unknown whether one demographic was more represented than another. Last, the perspectives obtained might have been affected by recall bias. Despite these limitations, to the best of our knowledge, this is the first multisite study that describes the unique perspectives of family members, friends, and HCP after discussing ACP preferences with AYA post completion of VMC. The concurrent voices captured here poignantly illustrate the shared sense of burden and benefit AYAs and all those who care for them experience trying to communicate during the complex journey at the end of life.

## Data Availability Statement

The raw data supporting the conclusions of this article will be made available by the authors, without undue reservation.

## Ethics Statement

The studies involving human participants were reviewed and approved by NIH Institutional Review Board: National Institutes of Health. The patients/participants provided their written informed consent to participate in this study.

## Author Contributions

SB, LW, and MP contributed to the conception and design of the study. SB, LW, JT, PM, CH, and KZ collected the data. SB organized the database and wrote the first draft of the manuscript. MG, SB, and AF performed the statistical analysis. LW wrote sections of the manuscript. All authors contributed to manuscript revision, read, and approved the submitted version.

## Conflict of Interest

The authors declare that the research was conducted in the absence of any commercial or financial relationships that could be construed as a potential conflict of interest.

## Publisher’s Note

All claims expressed in this article are solely those of the authors and do not necessarily represent those of their affiliated organizations, or those of the publisher, the editors and the reviewers. Any product that may be evaluated in this article, or claim that may be made by its manufacturer, is not guaranteed or endorsed by the publisher.
