# Supplementary material for: Adolescent and Young Adult Initiated Discussions of Advance Care Planning: Family Member, Friend and Health Care Provider Perspectives
Source: Front Psychol. 2022 Jun 8;13:871042. doi: 10.3389/fpsyg.2022.871042 (PMC9215331; doi:10.3389/fpsyg.2022.871042)
Supplement: Supplementary file 1 [file Data_Sheet_1.pdf]

Supplemental Data Sheet 1. Survey Questionnaire

1. Did participant NAME share what he/she had written in *Voicing My CHOiCES* with you?

☐ Yes (**Complete 1a. through 1e.)**
☐ No

If yes:

1a. Can you tell me what part of the advance care planning process NAME shared with you?
1b. What do you think this experience was like for NAME to share this with you?
1c. Do you think NAME would have had this conversation with you at this time if he/she were not participating in this study?

☐ Yes
☐ No

1d. Have there been any changes in your relationship since having this discussion?

☐ Yes, Please describe
☐ No

1e. Can you tell us what this experience was like for you?
2a. [*For the medical provider*] Have there been any change in NAME care or care plans since he/she completed these pages?

☐ Yes, Please describe
☐ No

2b. [*For the medical provider*] Have you tried to have any conversations with NAME prior to this about advance care planning?

☐ Yes, Please describe
☐ No

2c. Can you tell us what this experience was like for you?
2d. Do you have any recommendations about the best way to use a document like *Voicing My CHOiCES* within your practice?
